# Profound or Phlegmonoid Erysipelas

**Published:** 1858-07

**Authors:** George Niemeier


### ART. III.—*Profound or Phlegmonoid Erysipelas.* By George Niemeier, M. D.

I have observed this disease in the latter years frequently, it prevails in the winter months, when after a severe cold, suddenly a thaw sets in, and in the commencement of spring, say from January to April, just about the same time, when superficial erysipelas most commonly prevails; I at least never observed it in the middle of summer. The term profound indicates a deep seat, i. e. inflammation of the muscles, periosteum and bone; the term phlegmonoid a very high degree of inflammation.

*Ætiology.* Profound as well as superficial erysipelas, are constitutional blood diseases, caused by a miasmatic unknown poison; it belongs to the same genus as scarlet measles. In fact in Scarlet epidemics you will sometimes see individuals attacked with erysipelas, apparently replacing the scarlet process.

*Diagnosis.* Patients with inflammatory fever complain for two, three, or four days, of severe headache, uneasiness, general prostration, dirty tongue, vomiting or only inclination to throw up, no appetite, costive bowels, pain in the pit of the stomach. The pains in the limb are hardly noticed at first, until after a couple of days most severe, deep-seated pains are complained of; no swelling at first; in general, hardly any discoloration of the skin, until after some days, an œdematous swelling, generally of the whole limb, follows, with a tinge of a yellowish-gray color. Pains and swelling increase, with fever not more inflammatory, but rather of a typhoid character. The seat of the matter being deep, and the limb at the same time often considerably swollen, fluctuation is often not easy to be felt.

*Prognosis.* Generally not bad, but still a considerable time is required until the whole process is gone through; if fatal, death is caused either by pyæmia or by general dissolution of the system caused by the abundant formation of matter.

*Therapeutics.* If called to see the patient in the very outset of the disease, give an emetic; then open the bowels by tamarinds, salts or rhubarb and magnesia. As soon as the limb gets painful, apply leeches and hot fomentations. Support the system by wine, brandy, beef tea, quinine, increasing the doses when the inflammatory fever turns into typhoid. If, after the lapse of ten or twelve days, sufficient fluctuation does not distinctly show the seat of matter, put in the knife pretty deeply where the patient complained from the very first of the seat of the severest pain, and you will generally come to the right source of matter. If the erysipelas extending to the periosteum and bone, has caused caries, you may give with the very best effect, quinine combined with iodide of potass. in gradually increased doses, and as external application, hot aromatic fomentations, a wash of nitrate of silver, a solution of the tincture of iodine or of the nitrate of mercury. In children, especially of a scrofulous diathesis, you will generally succeed with these remedies, though you want time and good nourishing food.

